# Diseases of the Skin

**Published:** 1916-12

**Authors:** Malone Duggan


MALONE DUGGAN.

---

DISEASES OF THE SKIN. By Henry H. Hazen, A. B., M. D., Professor of Dermatology in the Medical Department of the Georgetown University, and sometime Assistant Dermatologist in the Johns Hopkins University. Published by C. V. Mosby Co.

All books presumably are written to fill some particular field, but not all books fulfill their purpose. The little book before us, however, fully measures up to its aims, and will prove to be a wonderful satisfaction to a large class of physicians. Dermatology is a subject very poorly understood by the average physician and he is often put to a loss to know how to handle the skin cases that he meets with in his general practice. This book will help the doctor to find himself, so to speak, and it will enable him to diagnose his case with unusual accuracy and saving of time, and also indicate the most modern line of both general and specific treatment. With this information, if needs be, he can consult more pretentious books with greater profit to himself. During the past three months in a base hospital service on the border the reviewer found the book to meet adequately his wants, and he has no hesitancy in highly recommending it to every physician who desires a book of this kind. MALONE DUGGAN.

